# Nanomedicine in HNSCC therapy-a challenge to conventional therapy

**DOI:** 10.3389/fphar.2024.1434994

**Published:** 2024-10-14

**Authors:** Chenyu Li, Yuan Fang, Sanchun Xu, Jingyuan Zhao, Deshi Dong, Shuai Li

**Affiliations:** ^1^ Department of Pharmacy, The First Affiliated Hospital of Dalian Medical University, Dalian, China; ^2^ School of Pharmacy, Dalian Medical University, Dalian, China; ^3^ Clinical Laboratory Center, Central Hospital of Dalian University of Technology, Dalian, China

**Keywords:** head and neck cancer, molecular pathogenesis, nano-drug, nanocarrier and delivery, cancer

## Abstract

Squamous cell carcinoma of the head and neck (HNSCC) is a difficult-to-treat cancer and treatment is challenging due to recurrence or metastasis. Therefore, there is an urgent need to explore more effective targeted therapies to improve the clinical outcomes and survival of HNSCC patients. The nanomedicine is emerging as a promising strategy to achieve maximal anti-tumor effect in cancer therapy. In this review, we summarize some important signaling pathways and present the current and potential roles of various nanomaterial drug-delivery formulations in HNSCC treatment, aiming to understand the pathogenesis of HNSCC and further improve the therapeutic efficacy of nanomaterial HNSCC. This article seeks to highlight the exciting potential of novel nanomaterials for targeted cancer therapy in HNSCC and thus provide motivation for further research in this field.

## 1 Introduction

Head and neck cancer (HNC) is a highly prevalent malignancy worldwide, with an annual incidence exceeding 600,000 cases. HNSCC originates from the mucosal surfaces of various anatomical sites, including the sinuses, lips, oral cavity, pharynx, and larynx (Johnson et al., 2020). The progression of HNSCC tumors follows a distinct sequence of stages, wherein the initial normal mucosa gradually undergoes hyperplasia, dysplasia, and ultimately transforms into cancer, accompanied by an accumulation of genetic mutations at each successive stage. Noteworthy etiological factors contributing to the progression of HNSCC encompass tobacco smoking, excessive alcohol consumption, and infection with human papillomavirus (HPV) (Galati et al., 2022). It is pertinent to note that HPV-positive patients devoid of a smoking or alcohol history typically demonstrate a more favorable prognosis.

In treating head and neck malignancies, a multimodal approach that includes surgery, radiotherapy, and chemotherapy is commonly used. Despite this, treatment outcomes are often unsatisfactory. Surgery can completely remove the local tumor, providing immediate results, but it may lead to significant postoperative changes in appearance and function (such as speech and swallowing) and is not suitable for all patients. The risks associated with surgical treatment are particularly high for patients in poor health or with advanced disease. Radiotherapy and chemotherapy, when combined, offer a non-invasive alternative that can preserve organ structure and function while treating cancer. However, this approach has numerous side effects, including secondary cancers and fibrosis, leading to a generally poor prognosis, especially in the later stages of the disease (Bhatia and Burtness, 2023).

Cisplatin is a commonly used first-line chemotherapy drug for HNSCC, working by binding to DNA and inhibiting tumor cell growth (Khera et al., 2023). It is often combined with 5-fluorouracil (5-FU). For patients resistant to cisplatin, paclitaxel and docetaxel are alternative options, though these drugs have significant side effects, such as nephrotoxicity and neurotoxicity, and prolonged use may lead to drug resistance. Radiation therapies like intensity-modulated radiation therapy (IMRT) and stereotactic body radiation therapy (SBRT) are precise but can still cause radiation-induced damage and recurrence (Cramer et al., 2019). The success rate of HNSCC treatment varies by disease stage; following chemoradiotherapy, early-stage patients have a 5-year survival rate of 70%–90%, while late-stage patients see a drop to 20%–30% (Cramer et al., 2019). Although these treatments are effective in early-stage HNSCC, there is a need for further optimization in treating late-stage or recurrent cases. Hence, there is an urgent need to enhance the specificity of drug therapy and improve the bioavailability of therapeutic agents. Nano-targeted drug delivery systems have emerged as a preferred strategy to effectively target a broad spectrum of tumors by prolonging drug circulation half-life, shielding the drug from the tumor microenvironment, thereby enhancing efficacy, and minimizing adverse effects (Calixto et al., 2014a). Furthermore, nano-targeted drug delivery holds promise in augmenting drug sensitivity and circumventing multiple resistance mechanisms in cancer cells (Wang et al., 2017). Nanomaterials showing promise in the targeted therapy of head and neck cancer include liposomes, mesoporous silica, and others. Among these, liposomes serve as effective drug carriers, enhancing the delivery of lipophilic drugs specifically to head and neck cancer cells. In head and neck cancer models, liposome-mediated plasmids have been used to target specific molecules such as the epidermal growth factor receptor (EGFR). This targeted approach has been shown to effectively inhibit the expression of these proteins, increase the rate of apoptosis in cancer cells, and suppress tumor growth. Additionally, mesoporous silica nanoparticles provide a versatile platform for drug delivery due to their large surface area and pore volume, which can be used to encapsulate and release therapeutic agents in a controlled manner, further contributing to the targeted treatment of head and neck cancer (Wu and Zhou, 2015). Consequently, in-depth comprehension of the molecular mechanisms driving the development of head and neck cancer is critical to identify promising targets for inhibiting tumor growth, as well as to explore nanomaterials suitable for targeted drug delivery to achieve precise treatment in this patient population. In this comprehensive review, we elucidate the pathogenesis of head and neck tumors and outline relevant therapeutic targets. We also delve into the advantages of nano-targeting systems and highlight potential nanomaterials for formulation development. Finally, we address the existing challenges in targeted drug delivery and provide our insights on the prospects of targeted therapies for head and neck cancer.

## 2 Molecular pathogenesis of HNSCC

Cancer typically originates from the accumulation of genetic and epigenetic alterations in genes associated with cancer-related signaling pathways. These changes enable the cells to proliferate indefinitely, evade apoptosis, and exhibit invasive and metastatic properties. The pathogenesis of non-hereditary NHSCC involves a complex, stepwise process characterized by mutations in specific genes and dysregulation of protein expression. These molecular alterations subsequently activate various signaling pathways that drive tumor initiation, progression, and metastasis (Leemans et al., 2011). Notably, HNSCC frequently perturbs crucial pathways such as p53/RB, EGFR, and PI3K/AKT/mTOR, along with other important molecules that serve as potential therapeutic targets. The proposed molecular mechanism of HNSCC carcinogenesis offers a novel approach to targeted therapy, facilitating the development of innovative treatment strategies and the advancement of personalized treatment modalities for individual patients.

### 2.1 The P53 and RB pathway

In HNSCC, mutations or loss of function of the TP53 and RB genes play a critical role in tumor initiation and progression (Leemans et al., 2011). The TP53 gene encodes the p53 protein, a crucial tumor suppressor that regulates the cell cycle, DNA repair, and apoptosis to prevent the proliferation of damaged cells. However, TP53 mutations are found in over 50% of HNSCC patients, often leading to the loss of normal p53 function. This results in ineffective DNA repair and apoptosis, which promotes tumor formation and progression and is associated with poor prognosis, treatment resistance, and higher recurrence risk (Robles and Harris, 2009; Poeta et al., 2008). Similarly, the RB gene encodes the Rb protein, another key tumor suppressor that controls cell proliferation by regulating the progression from the G1 to S phase of the cell cycle (Rozenberg et al., 2021). In HNSCC, RB function is often lost through mutations, methylation, or viral protein interactions, leading to uncontrolled cell cycle progression and excessive tumor cell proliferation. This is especially significant in HPV-associated HNSCC, where the HPV E7 protein binds to and inactivates the Rb protein, a mechanism particularly relevant in HPV-positive head and neck cancers (Xu et al., 2022).

Therefore, mutations or inactivation of TP53 and RB play a crucial role in the development and progression of head and neck cancer (Si et al., 2016). Targeting these genes or their associated pathways may improve patient outcomes and offer new treatment avenues. In head and neck cancers with TP53 mutations, nanomedicines can be designed to deliver small molecules, gene-editing tools (such as CRISPR/Cas9), or RNA interference molecules (such as siRNA) to repair or replace the inactive TP53 function (Deneka et al., 2022). This approach can restore normal p53 protein function, reactivating cell cycle regulation, DNA repair, and apoptosis pathways to inhibit tumor growth. For cancers with RB gene inactivation, nanomedicines can be used to deliver drugs or gene therapies to restore RB’s suppressive function or prevent abnormal cell cycle activation. For example, nanomedicines can deliver Cyclin-Dependent Kinase (CDK) inhibitors to block RB phosphorylation (el-Deiry et al., 1993), thereby restoring its inhibition of E2F transcription factors and preventing excessive tumor cell proliferation. By incorporating these drugs and gene therapies into nanocarriers, nanomedicines can achieve more efficient and precise drug delivery, overcoming some limitations of traditional treatments and showing significant potential in head and neck cancer therapy.

### 2.2 The EGFR pathway

**FIGURE 1:**
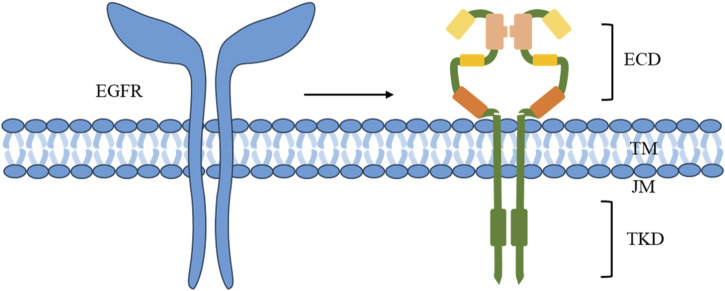
Components of EGFR.

The EGFR belongs to the ErbB/HER family of receptor tyrosine kinases (RTKs), which play a crucial role in HNSCC. While EGFR mutations and amplifications are infrequent occurrences in patients with HNC, they are highly expressed in over 80% of HNCSS cases, leading to significant impacts on overall and progression-free survival and are strongly correlated with an unfavorable prognosis (Solomon et al., 2018). EGFR is a transmembrane protein that comprises extracellular (ECD), transmembrane (TM), juxtamembrane (JM) fragments, and a tyrosine kinase domain (TKD) (Figure 1) (Li Q. et al., 2023). Perturbations in any of these components can result in oncogenic mutations, leading to abnormal expression and dysregulated signaling, along with altered responses to EGFR-targeting agents.

**FIGURE 2:**
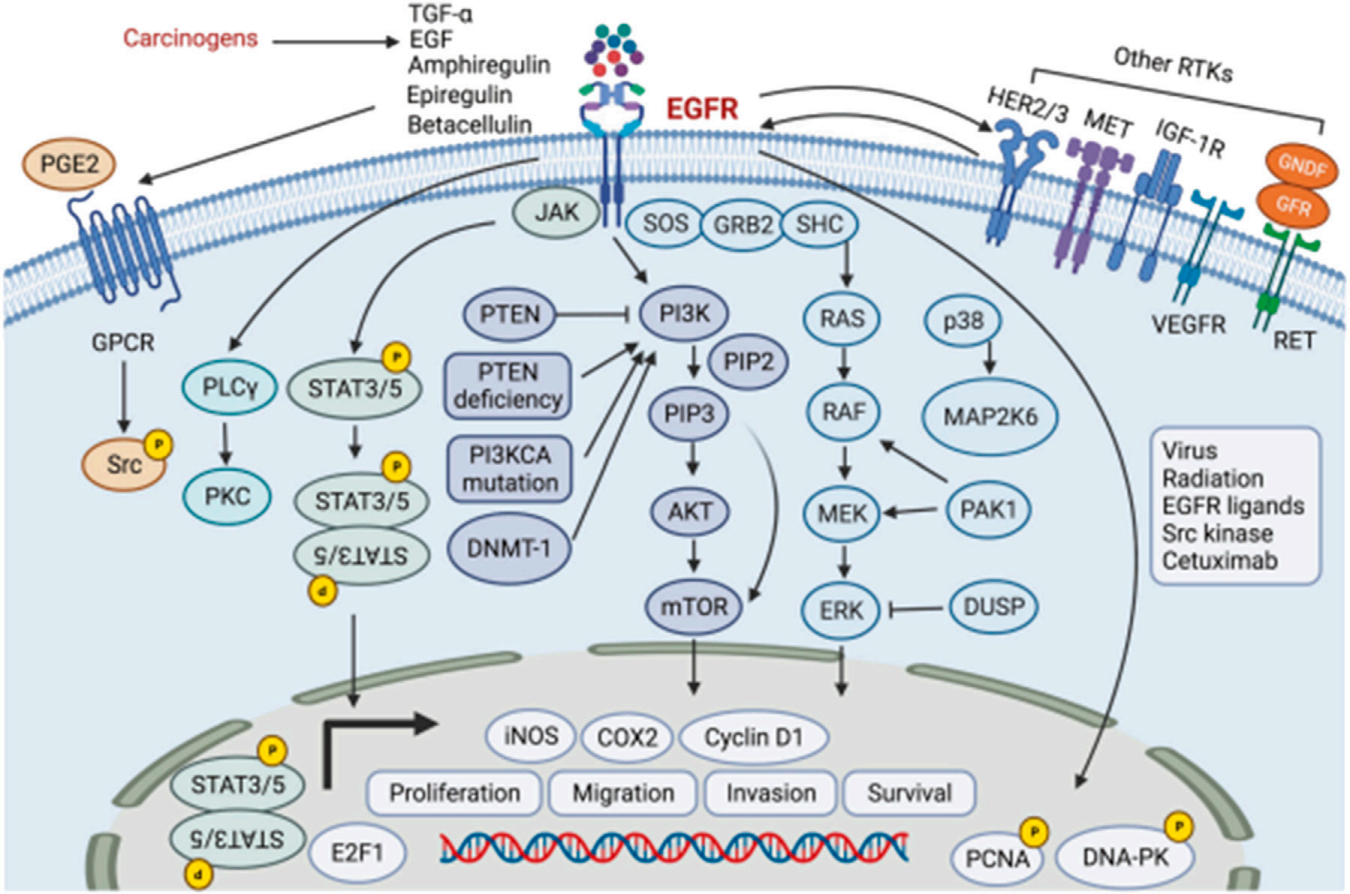
The EGFR signaling pathway, PI3K/AKT/mTOR pathway, MAPK pathway, STAT pathway, and MET pathway in head and neck cancer.

Disruptions in EGFR signals not only impact the binding process but also affect downstream signaling cascades (Figure 2) (Li Q. et al., 2023). In addition to its membrane-bound form, EGFR can translocate to the nucleus and undertake various functions. Nuclear EGFR acts as a transcriptional activator by interacting with STAT3 and E2F1, facilitating the transcription of genes such as cyclin D1, iNOS, B-Myb, and Aurora kinase A (Li et al., 2009). In head and neck cancer, EGFR can be activated through various mechanisms. This includes the autocrine or paracrine effects of EGFR ligands, such as increased production of amphiregulin and TGF-α in response to tobacco smoke. Additionally, activation of G-protein-coupled receptors (GPCRs) and the elevated levels of the GPCR ligand PGE2 also contribute to EGFR activation (Abo-El Fetoh et al., 2023). Researchers have also demonstrated that EGFR signaling induces the nuclear translocation of TSPAN8 by activating AKT kinase, which ultimately leads to direct phosphorylation of TSPAN129 at Ser8. Within the nucleus, phosphorylation of TSPAN8 enhances the chromatin occupancy of STAT3, thereby modulating the transcription of downstream oncogenes, such as MYC, BCL-2, and MMP-9. When EGFR undergoes mutations or amplification, the EGFR-AKT-TSPAN8-STAT3 axis becomes hyperactivated, resulting in an aggressive phenotype and poor prognosis of cancer (Lu et al., 2022). Therefore, the EGFR receptor is also an important target for the treatment of HCNSS.

### 2.3 The PI3K/AKT/mTOR pathway

The phosphatidylinositol-3 kinase (PI3K)/AKT and mammalian target of rapamycin (mTOR) signaling pathways play vital roles in numerous physiological and pathological processes, including cell proliferation, angiogenesis, anabolic responses, differentiation, and survival (Porta et al., 2014). In the context of HNC, the regulation of the PI3K/AKT and mTOR pathway is dysregulated, which can contribute to the development of various types of human cancers. Notably, the activation of this pathway is observed in more than 90% of both HPV-positive and HPV-negative HNCs (Marquard and Jücker, 2020).

PI3K is composed of a regulatory subunit (p85) and a catalytic subunit (p110), and activating mutations in the p110 catalytic subunit of PI3K are frequently identified in different cancer types, correlating with a poorer prognosis. AKT, a downstream effector of PI3K, exerts its influence on apoptosis through phosphorylation-mediated deactivation of proapoptotic proteins. For instance, it phosphorylates and inactivates BAD, a key regulator of cytochrome c release from mitochondria, as well as ASK1, a kinase regulator (Datta et al., 1997). Furthermore, AKT can activate mTOR through direct or indirect phosphorylation (LoPiccolo et al., 2008). mTOR, a serine/threonine kinase, is commonly activated in human cancers and plays a pivotal role in cancer development and treatment. It is crucial for PI3K/AKT signaling in HNC in response to tumor growth and proliferation.

mTOR exists in two distinct complexes, mTORC1 and mTORC2. mTORC1 phosphorylates and activates ribosomal protein S6 kinase (S6K) while inhibiting the eIF4E-binding protein (4EBP), resulting in enhanced metabolic enzyme activity and upregulation of metabolism-related transcription factors (Mossmann et al., 2018). On the other hand, mTORC2 primarily promotes metabolism by activating AKT (Saxton and Sabatini, 2017). Consequently, targeting the PI3K/AKT/mTOR signaling pathway represents a promising therapeutic approach for combating cancer, including HNC, paving the way for the development of effective antineoplastic agents.

### 2.4 The HGF/Met pathway

The Mesenchymal-epithelial transition (Met) factor represents a receptor tyrosine kinase encoded by the Met proto-oncogene. It binds to hepatocyte growth factor (HGF) ligands, thereby influencing cellular processes such as survival and proliferation. Activation of the HGF/Met axis has been extensively documented to stimulate cell proliferation, migration, invasion, and angiogenesis in HNSCC and other tumor types. Furthermore, it serves as a crucial mechanism contributing to resistance against anti-EGFR therapy (Knowles et al., 2009). Notably, MET protein overexpression is observed in more than 80% of HNSCC cases. In an *in vivo* model of HNSCC, the inhibition of Met leads to impaired cancer cell motility, reduced dissemination to lymph nodes, and an extended overall lifespan (Raj et al., 2022).

HGF serves as the exclusive ligand for Met and is overexpressed in approximately 50% of the stromal components associated with HNSCC. Interestingly, tumor cells do not secrete HGF themselves; instead, it is secreted by neighboring cells within the tumor microenvironment in a paracrine manner (Seiwert et al., 2009). *In vivo*, the HGF/Met signaling pathway facilitates various critical cellular processes, including growth, motility, invasive metastasis, angiogenesis, wound healing, and tissue regeneration (Liska et al., 2011). Consequently, the HGF/Met signaling axis presents an attractive target for HNC treatment. Approaches to target this pathway primarily encompass the use of monoclonal antibodies, tyrosine kinase inhibitors, and NK4 decoys, which act as antagonists of HGF (Hartmann et al., 2016). The specific principles are detailed in Table 1.

**TABLE 1:** HGF/Met targeting strategies.

Method	Principle
Monoclonal antibodies	Target either HGF or Met
tyrosine kinase inhibitors	Target the tyrosine kinase domain of Met
truncated soluble Met receptor	Act as a decoy for HGF and the competitive HGF antagonist NK4

### 2.5 The Wnt/β-catenin signaling and the notch pathway

The Wnt/β-catenin signaling pathway plays a crucial role in embryonic development, tissue regeneration, cell proliferation, and cell differentiation. Aberrant activation of this pathway is observed not only in HNC but also in various other cancer types (Clevers and Nusse, 2012). Extensive studies have revealed that the Wnt/β-catenin pathway promotes tumor cell proliferation, sustains a stem cell-like phenotype, facilitates tumor invasiveness in HNC, and is strongly associated with poor prognosis and increased mortality in patients with HNSCC (Yu et al., 2021).

Presently, at least 19 members of the Wnt family have been identified, such as Wnt1, Wnt3, Wnt3a, Wnt5, Wnt5a, among others. Different Wnt ligands exert specific effects on both canonical and non-canonical Wnt signaling pathways through specific interactions with distinct Wnt receptors and co-receptors (Xie et al., 2020), These canonical and non-canonical Wnt signaling pathways are both implicated in the progression of HNSCC. Notably, the canonical Wnt pathway, specifically through Wnt1, promotes invasion in oral squamous cell carcinoma (Rhee et al., 2002).

The Wnt/β-catenin protein signaling pathway and the Notch pathway are intricately intertwined and contribute to tumorigenesis in HNC (Collu et al., 2014). Notch1 plays a pivotal role in maintaining the properties of cancer stem cells and exacerbating tumor recurrence and metastasis. Mutations in Notch genes, leading to their inactivation, have been detected in 17%–26% of HNC cases, with Notch1 mutations being predominant. Furthermore, Notch receptors exhibit heightened expression levels in HNC and propagate signals through the transcriptional activation of target genes like HES1 and HEY1. Remarkably, 31.8% of HNSCC tumors display pronounced overexpression of HES1 and HEY1, signifying potential activation of the Notch signaling pathway in a substantial portion of HNSCC tumors (Sun et al., 2014). Additionally, deficiency in Notch receptors also triggers the upregulation of the δNp63 gene in HNC, thus fostering tumorigenesis.

### 2.6 The JAK/STAT pathway

In HNSCC, activation of the Janus-activated kinase (JAK)/signal transducer and activator of transcription (STAT) pathway, particularly involving STAT3 and STAT5, is closely associated with various oncogenic processes such as tumor cell proliferation, angiogenesis, immune evasion, treatment resistance, and unfavorable prognosis (Lai and Johnson, 2010). Initiation of this pathway occurs when the ligand binds to the cytokine receptor, resulting in JAK activation. Subsequently, JAK phosphorylates the STAT protein, facilitating its dimerization and subsequent nuclear translocation. Phosphorylated STAT3, localized within the nucleus, exhibits the ability to upregulate the expression of downstream target genes including cell cycle protein D1, survivin, and Bcl-xL. These genes play crucial roles in tumor cell proliferation, angiogenesis, and immune evasion (Wang et al., 2004). Excessive activation of STAT3 is significantly correlated with poor prognosis, resistance to standard therapies, and immune escape, thus presenting a promising target for HNSCC treatment. Some studies suggest that targeting this pathway may be particularly beneficial in HPV-negative HNSCC cases (Gaykalova et al., 2015). Moreover, evidence implies that several members of the protein tyrosine phosphatase receptor (PTPR) family may contribute to tumor suppression by dephosphorylating STAT3 (Veeriah et al., 2009).

### 2.7 The Hippo pathway

In HNSCC, dysregulation of the Hippo pathway significantly impacts tumor development and progression. The Hippo pathway controls cell proliferation and organ growth by inhibiting the transcriptional co-activators YAP (Yes-associated protein) and TAZ (transcriptional co-activator with PDZ-binding motif) (Santos-de-Frutos et al., 2019; Faraji et al., 2022). Under normal conditions, activation of the Hippo pathway prevents YAP and TAZ from translocating to the nucleus, thereby inhibiting abnormal cell proliferation and tumor formation. However, in HNSCC, the Hippo pathway is often disrupted, leading to excessive activation of YAP and TAZ. This disruption is primarily due to the inactivation of the tumor suppressor gene FAT1 and the abnormal activation of the PI3K pathway (Martin et al., 2018). FAT1 mutations or deletions result in loss of Hippo pathway function, while activation of the PI3K pathway further impairs Hippo signaling and enhances YAP and TAZ activity (Ando et al., 2022). This overactivation is closely associated with tumor grade, stage, lymph node metastasis, and poor prognosis. Therefore, therapeutic strategies targeting the Hippo pathway, such as direct inhibition of YAP/TAZ, development of inhibitors targeting FAT1 and PI3K, and the use of BET inhibitors, have emerged as promising approaches for treating head and neck cancer.

In conclusion, the development of HNC is regulated by a complex interplay of multiple signaling pathways. While significant progress has been made in understanding these mechanisms, further exploration is warranted to uncover additional avenues for targeted therapy. This includes identifying novel targets within these pathways and developing innovative therapeutic strategies to improve the outcomes of patients with HNC.

## 3 Nanomaterials for targeting HNC

Traditional drug delivery methods have weak targeting capabilities, causing drugs to distribute throughout the body, which increases side effects and toxicity. Additionally, these methods suffer from poor drug stability and require frequent dosing (Song et al., 2021). In contrast, nanocarrier-based targeted delivery systems concentrate drugs at specific disease sites through precise targeting mechanisms, improving therapeutic efficacy and reducing side effects (Zhao et al., 2020; Majumder et al., 2019). Nanocarriers can also protect drugs, extend their half-life, control drug release, and optimize pharmacokinetic properties. These systems are multifunctional and can cross complex biological barriers, showing great promise in the treatment of diseases, especially cancer (Hossen et al., 2019). For instance, RL and their team successfully designed the NPF@DOX nano drug delivery system, which demonstrated superior targeting accuracy towards oral squamous cell carcinoma compared to doxorubicin (DOX) alone. Moreover, under near-infrared radiation, the system achieved a notable photothermal conversion efficiency of 52.48%. The heat generated by NPF@DOX further facilitated the localized release of DOX and triggered apoptosis through a pH-sensitive mechanism (Li et al., 2023b).

**FIGURE 3:**
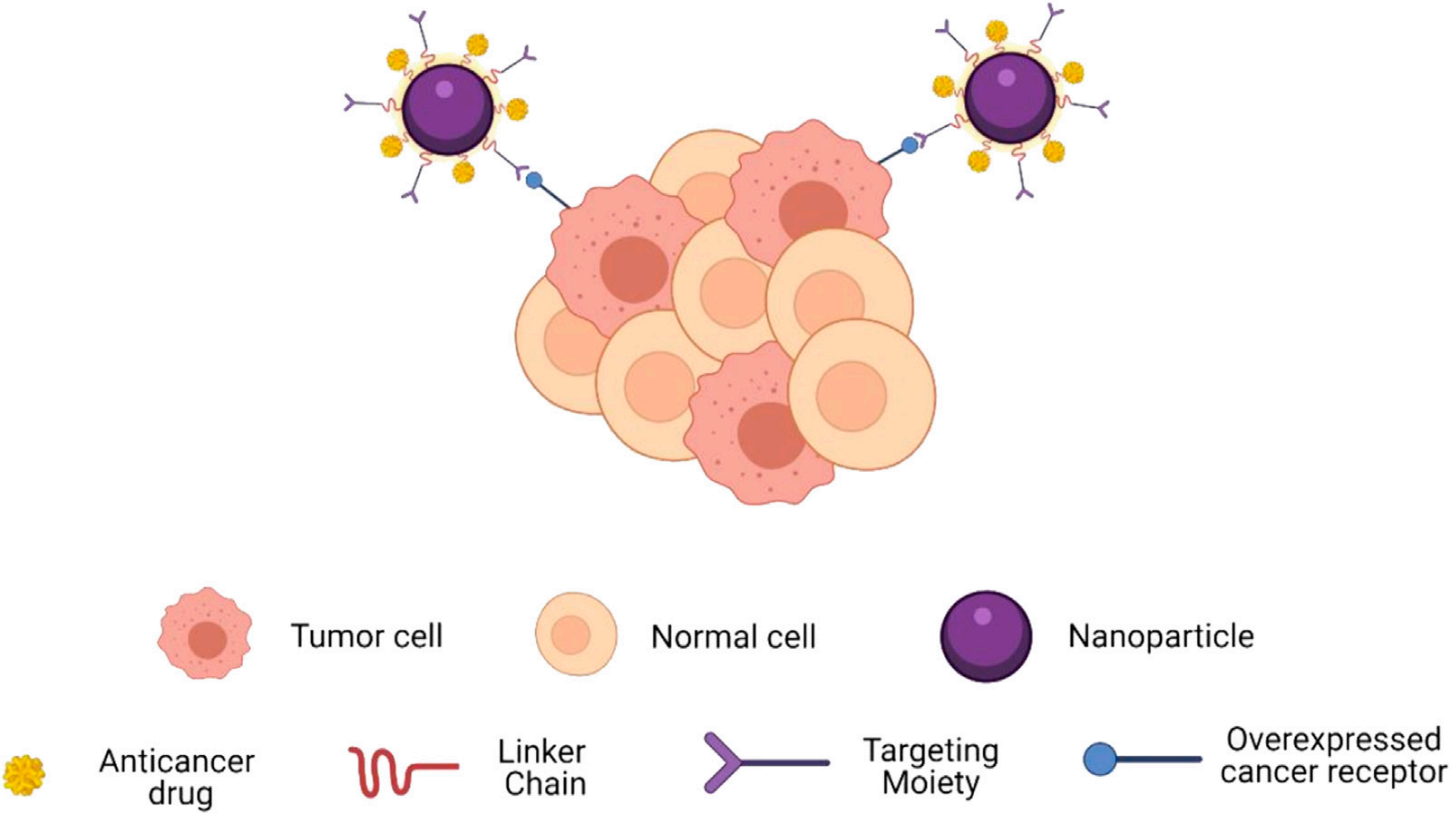
Schematic showing how targeting moieties provide active targeting. Targets enable spe-cific binding to overexpressed receptors on cancer cells.

Nanoparticles (NPs) have garnered substantial attention in the realm of biomedical materials due to their unique physicochemical attributes, diminutive size, and versatile functionality (Doane and Burda, 2012; Nicolas et al., 2013; Arvizo et al., 2012). Targeted therapy necessitates the selective enrichment of nanomaterials within tumor tissue, a feat accomplished through two primary mechanisms: passive targeting and active targeting. Passive targeting capitalizes on the Enhanced Permeability and Retention (EPR) effect to enhance drug concentration at the tumor site. During the course of tumor development, there is a rapid emergence of aberrant blood vessels that exhibit heightened susceptibility to leakage, resulting in impaired lymphatic drainage from the tumor. This phenomenon facilitates the accumulation of therapeutic agents within the tumor microenvironment (Wang and Thanou, 2010). In contrast, active targeting leverages affinity ligands to specifically bind NPs to antigens or extracellular matrix proteins within the tumor tissue (Prabhakar et al., 2013) (Figure 3) (Ruiz-Pulido et al., 2021). Furthermore, to augment drug accumulation and release within the tumor tissue while mitigating side effects, active targeted drug delivery systems can be meticulously engineered based on principles of blood circulation and osmotic control. The genesis of targeted NPs can be traced back to the 1980s when the utilization of monoclonal antibodies for the recognition of antigens on target cells, enabling the selective accumulation of drugs within tumor cells, was first reported (Leserman et al., 1980).

To date, researchers have extensively investigated and utilized a diverse array of nanomaterials. Noteworthy examples include carbon-based nanoparticles such as carbon nanotubes, graphene, and fullerenes (Chung et al., 2013), silica-based NPs (Bitar et al., 2012), semiconductor NPs, metal and metal oxide NPs (e.g., gold, silver, and iron oxides) (Arvizo et al., 2012), organic NPs (e.g., liposomes, polymer NPs, hydrogels, and dendritic polymers), and protein NPs (Calixto et al., 2014b). Nanomaterials offer an effective platform for the focused distribution of drugs, genes, proteins, and other bioactive substances, with significant potential in treating cancer. In addition to functioning as specific transporters for therapeutic drugs, a number of inorganic nanoparticles (including gold NPs, semiconductor NPs, and magnetic NPs) due to their distinct physical characteristics, can also be proficiently employed in the diagnosis, detection, and treatment of tumors through photoacoustic imaging, photothermal or photodynamic therapy, and hyperthermia therapy.

### 3.1 The inorganic material

#### 3.1.1 Au nanoparticles

Au nanoparticles exhibit remarkable biocompatibility and low toxicity, rendering them suitable carriers for chemotherapeutic drugs in the context of targeted tumor therapy through passive or active targeting strategies. The distinctive optical properties of gold nanoparticles arise from their localized surface plasmon resonance (LSPR), making them amenable to exploitation for *in vivo* optical imaging. A portion of the energy absorbed by gold nanoparticles is emitted as scattered light, while the remainder undergoes non-radiative decay, generating heat. This phenomenon forms the basis of numerous applications of Au nanoparticles in optical imaging (Capek, 2017). The LSPR effect occurs when the unbound charges on the surface of Au nanoparticles oscillate in response to the electromagnetic field. This results in optical absorption levels substantially exceeding those achievable with organic dyes (Jain et al., 2006). Within the wavelength range of the “biological window” (650–1100 nm), where blood and tissue attenuation are minimized, light-absorbing nanoparticles enable high-contrast visible imaging, thereby facilitating optical imaging (Weissleder, 2001). Absorption spectra of nanoparticles can be tuned by their geometry and size (Prodan et al., 2003), e.g., different types such as Au nanorods, Au nanocages, etc., as well as the effect of aspect ratios of different sizes on absorbance and scattering, etc. (Huang et al., 2009). Additionally, photoacoustic imaging using AuNPs is suitable for detecting different types of cancers. The plasma Au nanoparticles are capable of indicating the presence of a tumor either by accumulating in it through enhanced permeability and retention effects or by being functionalized for accumulation through receptor-mediated binding. Targeting and detecting tumors using gold nanoparticles is possible with surface-enhanced Raman scattering (SERS). The researchers systemically injected gold nanoparticles coupled with ScFv antibodies, which specifically bind to EFGR, into nude mice carrying EGFR-positive head and neck tumors (Tu686). They found that the ScFv-coupled Au nanoparticles were able to accurately target the tumors *in vivo* (Qian et al., 2008).

Au nanoparticles that scatter near-infrared light can also be used as enhancers for optical tomography. Due to coupled equipartitioned exciton resonance, aggregated gold nanorods can also exhibit significant NIR absorption, which, when used to fabricate nanostructures with NIR photothermal properties, can be used as both an ideal probe for photothermal therapy and as a contrast agent in PA imaging (Yang et al., 2009). It has been demonstrated that gold particles can be heated by shortwave radiofrequency fields to selectively treat deep tissues (Gannon et al., 2008). In conclusion, gold nanoparticles have the potential to be useful in cancer imaging, spotting tumors, delivering drugs, and carrying out photothermal and photodynamic therapies for cancer treatment.

Au nanoparticles have emerged as promising candidates for the treatment of HNC. Their unique physicochemical properties, such as high surface area-to-volume ratio, biocompatibility, and ease of surface functionalization, enable them to interact with biological systems effectively (Kesharwani et al., 2023). Au nanoparticles can induce cell death in HNC cells through various mechanisms, including apoptosis, autophagy, and necrosis. Moreover, Au nanoparticles can enhance the efficacy of radiation therapy and chemotherapy when used in combination. Additionally, Au nanoparticles can serve as contrast agents for imaging and as carriers for targeted drug delivery, further enhancing their potential in HNC treatment (Fan et al., 2020). Despite these promising attributes, more research is needed to fully understand the mechanisms of Au nanoparticles action in HNC and to optimize their clinical application for improved patient outcomes.

#### 3.1.2 Graphene

In recent years, graphene has been widely used in photothermal tumor therapy (PTT) and targeted drug delivery (Zhou et al., 2021). Graphene-based drug carriers can extend the circulation time of the drug, increase the drug loading and control the release of the drug (Zhou et al., 2014), and also enhances drug accumulation at the tumor site through enhancement of the EPR effect (Liu et al., 2013; Sattari et al., 2021). Meanwhile, Graphene nanoparticles created through coupling proteins, fluorophors, pharmaceuticals and protective coatings with functional groups on the graphene surface have the potential to improve drug delivery (Yang et al., 2013). For instance, the introduction of hydrophilic portions (e.g., hydroxyl and carboxylic acids) to graphene oxide nanoparticles (NGO) enables Effective distribution in water-based solutions, decreases toxicity, and enhances biocompatibility, indicating potential as a drug carrier material (Kiew et al., 2016). In addition, polyethylene glycol (PEG) ated nanographene oxide has better physiological stability and therapeutic effect. The researchers used NGO-PEG to bind the water-insoluble aromatic drug camptothecin (CPT) analog SN38 through π-π stacking and found that NGO-PEG-SN38 has a stronger cancer cell killing ability than the water-soluble prodrug irinotecan (CPT11) (Liu et al., 2008). In addition to being used as a drug carrier,due to the unique light absorption properties of NGO, it can convert near-infrared light into thermal energy for PTT. As the NGO accumulates and absorbs near-infrared light within the tumor, it can elevate the temperature of the surrounding area, ultimately inducing apoptosis of cancerous cells (Chen et al., 2016). Doxorubicin (DOX) is a fluorescent anticancer medication that demonstrates excellent therapeutic efficacy and activity against solid tumours. Regrettably, its clinical use is restrained owing to the serious side effects it produces, among which is cardiotoxicity (Primeau et al., 2005). Li R et al. developed a novel drug delivery system (NPF) that targets tumors and can transport DOX by conjugating FAP-targeting peptide with NP. This approach was combined with photothermal therapy, which prevents systemic toxicity and overcomes drug resistance by solving the issue of insufficient drug accumulation caused by tumor localization problems. This study demonstrated the effectiveness of targeted FAP chemotherapy in combination with PPT for *ex vivo* and *in vivo* treatment of oral squamous cell carcinoma (Li et al., 2023c).

#### 3.1.3 Porous silicon nanoparticles

Porous silicon nanoparticles (pSiNPs) have attracted much attention in biomedicine due to their low toxicity and potential for minimally invasive and focal therapies avoiding conventional side effects (Vivero-Escoto et al., 2012). pSiNPs have a non-toxic, highly active surface area (up to 1000 m2/g, allowing other molecules to modify Si through various surface chemical reactions such as hydrogenation, silanization, and hydrocarbonization) and porous nanostructures (Drug, Probe, Enzyme, Protein, Antibody, siRNA loadable etc.), which facilitate its targeted and controlled drug release into cancer cells (Park et al., 2009; Wang et al., 2014; Li et al., 2018). In addition, the optical properties of silicon nanostructures, i.e., intrinsic photoluminescence, offer an alternative for bioimaging, accompanied by better biocompatibility and biodegradability and lower toxicity compared to semiconductor quantum dots (Erogbogbo et al., 2008; Gu et al., 2010).

PSiNPs have emerged as promising candidates for the treatment of HNC due to their unique properties. These nanoparticles possess a high surface area, tunable pore size, and biocompatibility, making them suitable for drug delivery applications. PSiNPs can be loaded with various therapeutic agents, including chemotherapeutic drugs, small molecules, and nucleic acids, allowing for targeted and controlled release at the tumor site. Additionally, the porous structure of PSiNPs enables the co-delivery of multiple drugs or imaging agents, further enhancing their therapeutic efficacy. PSiNPs have shown promising results in preclinical studies, demonstrating their potential for use in the treatment of HNC. Further research is needed to optimize the design and formulation of PSiNPs for clinical translation in HNC therapy.

### 3.2 The organic material

#### 3.2.1 Biodegradable polymers

Owing to their ability to biodegrade, sustain drug release, boast nanometer size, exhibit biocompatibility and bioactivity, lack toxicity, possess long cycle times, avoid immunogenicity and can accommodate various active molecules like drugs, oligonucleotides and peptides, polymer nanoparticles hold promise as candidates for cancer treatment (Kumari et al., 2010). Polymer nanoparticles used as carriers for targeted delivery of drugs are typically Polymeric nanoparticles used as carriers for targeted drug delivery are typically between 10 and 1,000 nm in size and consist of drug-protecting polymers and copolymers, with a core-shell structure consisting of a polymeric matrix loaded with hydrophobic drugs on the inside and an outer surface of hydrophilic polymers. This method improves dural stability and reduces immunogenicity and phagocytosis of the nanoparticles. It also improves the efficacy of the reticuloendothelial system (Discher and Eisenberg, 2002). Polymers can be categorised as synthetic and natural polymers. Synthetic polymers include N-(2-hydroxypropyl)-methacrylamide copolymers, polyethylene glycol (PEG), poly-l-glutamic acid, poly (lactic acid) (PLA), poly-d, l-propylene glycol-co-glycol (PLGA), polysebacic acid, poly (acrylic acid) and others. Natural polymers such as heparin, chitosan, dextran, gelatin, etc., whose nanoparticles have the advantages of reduced drug side effects, sustained drug release, and increased residence time, are preferred for the delivery of a variety of active ingredients such as DNA, drugs, oligonucleotides, and proteins. For example, studies have shown that chitosan solution (CS)-modified polycaprolactone (PCL) synthesis of composite nanoparticles can improve the efficacy of 5-fluorouracil in cell lines for the treatment of HNC (de Lima et al., 2021).

Typically, self-assembly of copolymers of hydrophobic polymers (PLA, etc.) and hydrophilic polymers (PEG, etc.) is used to synthesise polymer nanoparticles for targeted drug delivery. Synthesis methods include nanoprecipitation (solvent displacement method) (Jung et al., 2000), oil-in-water (O/W) emulsification-solvent evaporation (single emulsion) and water-in-oil-in-water (W/O/W) emulsification-solvent evaporation and salting out (Zhuang et al., 2017). Furthermore, novel tecHNiques for fabricating polymeric nanoparticles, such as supercritical tecHNology, electro-spraying, premix membrane emulsification, and aerosol flow reactor methods, are presently being examined. A recent review offers further elaboration on these methods (Moya-Lopez et al., 2022).

Polymer nanoparticles have the following capabilities: (1) release of the drug at a predetermined experimental rate over an extended period of time, (2) preferential delivery of the drug to the target site with the ability to control the rate of release, (3) maintenance of appropriate therapeutic drug concentrations in both the circulation and tissues, and (4) protection of the drug (small molecule, protein, nucleic acid or peptide) from hepatic inactivation, enzymatic degradation and rapid clearance *in vivo* (Kamaly et al., 2012). In addition, by modifying the NP surface with polyethylene glycol (PEG) polymers, it is possible to prolong circulation time by preventing non-specific binding of the NP surface to blood components and by reducing the rapid uptake and clearance of mononuclear phagocytosis system (MPS) cells *in vivo* (Allen and Chonn, 1987). Polymeric NPs encapsulate a variety of drugs and drug molecules are released in a regulated manner by diffusion through the polymer matrix or by differential surface and bulk erosion rates of the particles. By carefully selecting the composition of polymer nanoparticles and incorporating specific targeting ligands into the polymers, drug accumulation in tumor tissues can be increased and efficacy enhanced (Seow et al., 2007).

#### 3.2.2 Protein material

Protein particles have been well studied and used in nanomedicine because they are easy to obtain, can be made again and again, interact well with the body and have many useful components. Depending on the source, protein nanomaterials for targeted delivery of drug carriers can be classified into animal proteins, plant proteins, and protein cages (Kianfar, 2021). Specific classifications and functions can be found in Table 2.

**TABLE 2:** Classification and function of protein nanoparticles.

Source	Protein nanoparticles	Functionality
Animal protein	Gelatine	The active moiety can be chemically modified directly or indirectly using different connectors, can be used as a carrier for targeted delivery of drugs, and can load large amounts of drugs onto the carrier
	Collagen	Small size, high surface area, good absorbency and dispersion in water to form a stable clear colloidal solution and can be used as a drug carrier for long sustained release of antimicrobials and steroids
	Casein	Cost effective, easy to access, stable, protection of sensitive goods, use as nanomedicine delivery
	Filaggrin	Protects proteins and peptides and increases the duration of active release of these compounds
	Elastin	Attaches multiple drug molecules and temperature-sensitive
	Albumin	
Plant protein	Corn Alcohol Protein	Good biodegradability, high hydrophobicity, special brick-like structure,and can be used as a controlled release delivery system for hydrophobic drugs and hydrophilic molecules such as heparin, 5-fluorouracil and adriamycin
	Maltitol protein	Using as a nanoparticle drug release Control system for hydrophobic and amphiphilic compounds (e.g., vitamin A, vitamin E, amoxicillin)
	Agglutinin	Using as a carrier for targeted drug delivery to induce apoptosis in cancer cells
	Soy protein	----
Protein cage	Viral protein cage	Targeted delivery of chemotherapy drugs or radioactive substances to tumour tissue
	Non-viral protein cage	

Among protein nanomaterials, albumin has a significant role in targeted drug delivery. It is a crucial protein in blood plasma with various important physiological functions. With its biocompatibility and biodegradability, multiple and functional groups, and high structural stability, it can serve as a drug transporter for treating cancer and viral diseases. Albumin can gather in cancerous tissue and discharge therapeutic substances by either passively targeting or actively stimulating targeting. For example, albumin can attach itself to a receptor on the outer layer of vascular endothelial cells called albobandine or glycoprotein 60 (which is involved in endocytosis) and accumulate there. Then, the drug-albumin mixture moves from the inner surface of the endothelial cell to the space between tissues, where it binds to extracellular matrix proteins known as SPARK and releases the drug for a prolonged period in the vicinity of the cancer cell. SPARK proteins are overexpressed in several cancers and can also serve as targets for targeted delivery of drugs (Elzoghby et al., 2012). In addition, albumin nanoparticle-loaded paclitaxel has been used to treat metastatic cancers (Ensign et al., 2012).

#### 3.2.3 Liposome

Liposomes are the most extensively utilized drug carriers and the most notable nanoparticle drug delivery method licensed by the FDA for clinical use in nanoparticle-mediated drug delivery. Liposomal nanoparticles (generally 100–500 nm in diameter) are unilamellar or multilamellar nanovesicles generated by phospholipid self-assembly (Phospholipids consist of a polar head made of phosphate and a hydrophobic tail composed of lipids).

**FIGURE 4:**
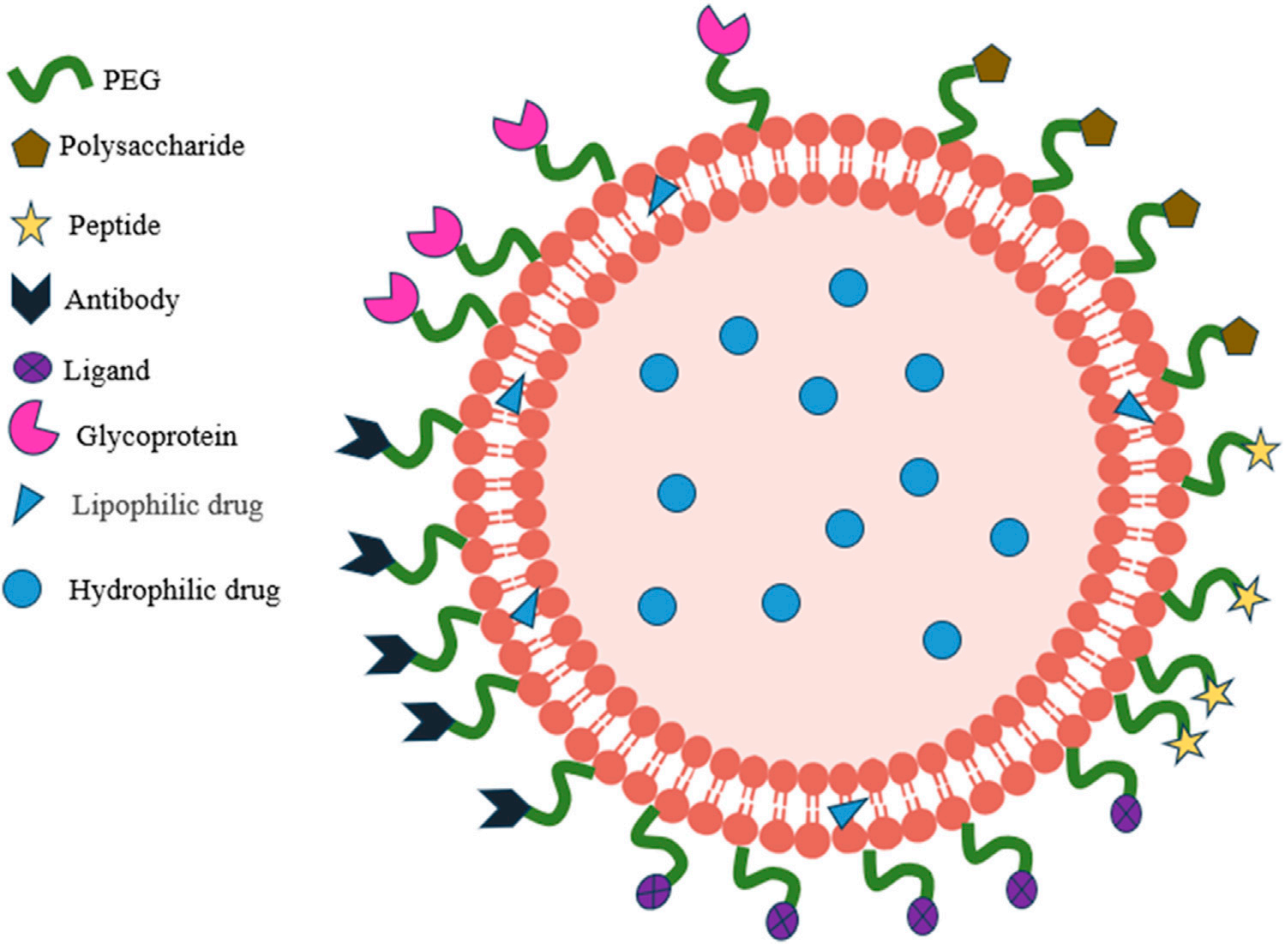
Schematic representation of target-modified liposomes.

**FIGURE 5:**
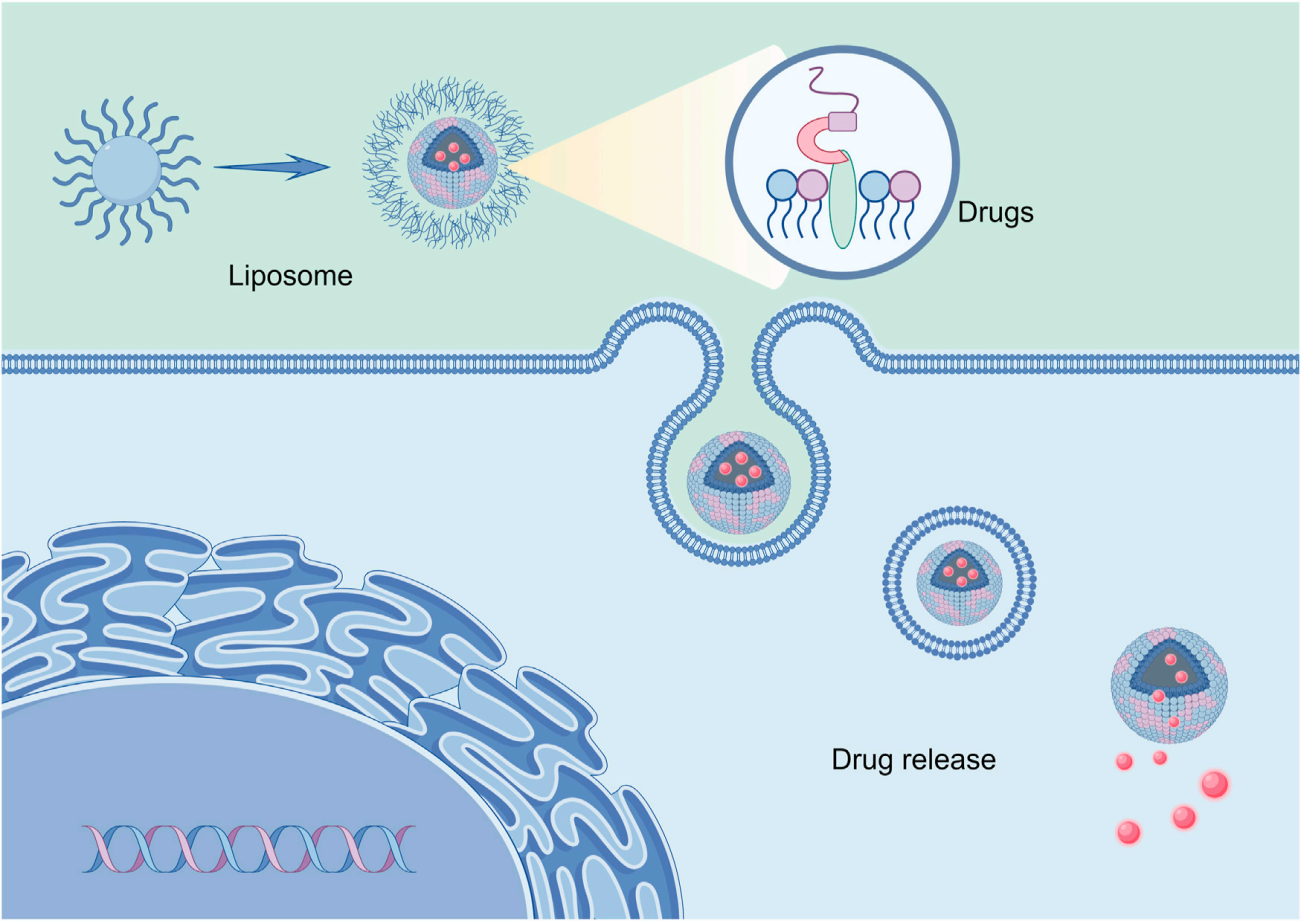
Liposome-encapsulated drug targeting into tumor cells.

In an aquatic setting, hydrophobic tails will self-orientate, resulting in a sphere with an aqueous center and a lipophilic bilayer membrane. Liposomes possess biocompatibility and biodegradability at specific pH and temperature thresholds, and their composition can be modified via changes made to the lipid. Moreover, liposomes can undergo numerous modifications to increase their efficacy as a targeted drug delivery method. For instance, when a polyethylene glycol (PEG) layer adheres to the surface of liposomes, it creates “invisible” liposomes. This protects the nanoparticles from renal clearance and extends the circulation time (Nag and Awasthi, 2013). In addition to conventional stealth liposomes, site-specific targeting liposomes can be created by combining targeting ligands such as antibodies, peptides, glycoproteins, oligopeptides, polysaccharides, growth factors, folic acid, carbohydrates, and receptors, which can increase the accumulation of drug-loaded liposomes in tumor tissues or cells (Song et al., 2009; Takara et al., 2010; Shmeeda et al., 2010), Shown in Figure 4. In addition, the basic delivery process of liposomes is shown in Figure 5. At the same time, it was shown that liposomes modified with dual ligands (e.g., MAN and TF) showed more significant therapeutic effects than liposome surfaces modified with drugs alone, drug-carrying liposomes, or mono ligands (Ying et al., 2010). In addition, stimulus-sensitive liposomes made in the presence of stimulants such as pH, light, magnetism, temperature, and ultrasound can control the release of drugs, proteins, and genes by regulating the environment. Studies have shown that exposure to ultrasound of liposomes loaded with perfluorocarbon gas triggers the delivery of drugs and genes through the cell membrane pore into the cytoplasm of target cells (Liu et al., 2006).

Loading drugs into liposomes is a significant hurdle encountered in the production of drug delivery carriers. Generally, drugs are doped through either passive or active methods (Sercombe et al., 2015; Zununi Vahed et al., 2017). The drug is dissolved in the organic phase with a phospholipid mixture by passive incorporation. Solvent evaporation and film formation follow, leading to the encapsulation of the drug in a water core (hydrophilic drug) or lipid bilayer (hydrophobic drug) by liposomes through water and action. The medication is loaded into the produced liposome by active incorporation by producing a transmembrane pH gradient (i.e., the pH of the buffer environment within and outside the liposome is different) and the drug subsequently forms a complex with the “trap” (ammonium sulfate, cyclodextrin, etc.) in the liposome (Alyane et al., 2016), and thus control the rate of drug release from liposomes (Yang et al., 2018). Additionally, liposomes can be altered with proteins or small molecules (antibodies, aptamers, peptides, carbohydrates, glycopolymers, vitamins, etc.) to create targeted bioconjugates that deliver medications to particular cell types or regions. Liposomes can be employed as complex agents for simultaneous tissue/cellular imaging and drug administration since they can be combined with a wide range of medications and small compounds. Along with the methods mentioned above, drugs can also be added to liposomes using a combination of tecHNiques, such as passive incorporation of drugs during or after liposome synthesis, covalent coupling during synthesis, etc., to carry multiple drugs or accomplish multiple objectives (trigger drug release, regulate drug activity, target specific cells, etc.) (Almeida et al., 2020). By establishing a xenograft HPV-positive HNC mouse model, Liyona et al. demonstrated that targeting with an anti-EGFR monoclonal antibody not only enhances the *in vivo* specific uptake of LNPs in cancer cells, but also enhances the therapeutic efficacy by stimulating anti-tumor activity (Kampel et al., 2021). In the treatment of head and neck cancer, the researchers evaluated intraoperative liposome therapeutic radionuclides by nude mouse xenograft-positive surgical margin model, and due to excellent tumor suppression and minimal side effects, the intraoperative liposome therapeutic radionuclide may play a role in the management of positive margins in advanced HNSCC surgery (Wang et al., 2008).

#### 3.2.4 Dendritic polymers

Dendritic polymers are named after trees due to their tree-like appearance. They are tiny, artificial polymers made up of many branched monomers spreading out from a core. Due to their central cavities, adaptability, simplicity of surface modification, well-defined round shapes, predictable weight, lack of immune response, water-soluble nature and lack of size limitation, dendritic polymers make excellent candidates for drug delivery. However, using them is limited due to biocompatibility and biodistribution issues, even though they can be combined with various substances like image-forming agents, drugs, and targeted ligands to create multifunctional drug delivery systems (Cho et al., 2008).

In HNC therapy, dendritic polymers can be tailored to carry various therapeutic payloads, including chemotherapeutic drugs, nucleic acids, and imaging agents. Their multifunctional nature allows for the conjugation of targeting ligands, such as antibodies or peptides, which can enhance their specificity for cancer cells while minimizing off-target effects. Preclinical studies have demonstrated the efficacy of dendritic polymers in delivering therapeutic agents to HNC tumors, resulting in improved antitumor activity and reduced systemic toxicity. However, further research is needed to optimize their formulation, pharmacokinetics, and toxicity profile for clinical translation.

#### 3.2.5 Hydrogel

Hydrogels are networks of hydrophilic polymer chains suspended in water. After swelling, the hydrogel dissolves and releases the drug through the interstices of its network. With superior performance and advantages such as easy formulation, targeted injection, biodegradability and sustained release, hydrogels have attracted much attention in targeted drug delivery. The barrier of poor solubility observed with many chemotherapeutic drugs may be overcome by topical injection of hydrogel formulations, providing continuous and controlled drug delivery within the tumor site, with minimal side effects from systemic exposure to the drug. This results in reducing the amount of drug required and increasing the amount reaching the tumor site (Norouzi et al., 2016). Hydrogels are divided into two categories: ordinary hydrogels and smart hydrogels based on how they react to external stimuli. Smart hydrogels are sensitive to environmental variables like pH, temperature, and photovoltaics and change their shape and characteristics as a result. This allows the loaded drug to release in a controlled manner (Fan et al., 2019). Through intratumor injection of NDIMH (nano-DOX-ICG MMP responsive hydrogel) and 808 nm NIR irradiation in nude mice bearing human HNSCC-15 xenografts, the researchers examined the synergistic anti-tumor efficacy and biosafety of hydrogel therapy *in vivo*, indicating that this approach may be a promising chemo-phototherapy alternative to HNSCC (Wang et al., 2019).

## 4 The challenge of nano-targeted systems

Compared to conventional drug dosage forms, nanoformulations present enhanced prospects for treating HNC and other diseases, owing to their distinct size, shape, and diverse material properties. Beyond facilitating targeted drug delivery, nanoformulations also provide controlled drug release and improved drug stability, significantly enhancing therapeutic efficacy. Extensive research on nanopreparations has yielded promising outcomes in both animal models and *in vitro* studies, although most of these findings are predominantly at the research stage, with limited clinical applications. However, significant progress has been made in the research of using nanomedicines for the treatment of head and neck cancer. Guo et al. designed and synthesized three different forms of nanopoly (dopamine) (nPDA) nanomaterials, loading cisplatin into them to create the nPDA-cis (a nanomedicine carrier system for cisplatin) (Guo et al., 2024). Additionally, Verena et al. developed a novel nanomedicine HFt-MP-PAS40-Dox using human ferritin heavy chain (HFt) and DOX (Damiani et al., 2017). Compared to free drugs, these formulations exhibit stronger targeting capabilities for head and neck cancer tumors, reduced side effects, and can be administered safely at higher doses, thereby demonstrating superior efficacy in controlling HNSCC malignancies. It is noteworthy that the emergence of personalized medicine offers potential for targeted drug delivery systems to assume a vital role. The advent of precision medicine has mitigated the impact of patient heterogeneity, enabling the development of personalized treatment plans tailored to individual patients, thereby optimizing the effectiveness of nanomedicines.

Nanomedicines show significant potential in the targeted treatment of head and neck cancers but face several challenges. Firstly, biological barriers such as the tumor microenvironment and the blood-brain barrier limit the effective penetration and distribution of nanoparticles. Additionally, the materials used in nanomedicines may cause toxicity or immune reactions, impacting their biocompatibility and safety. Moreover, ensuring the stability of nanoparticles and controlling drug release are difficult, and targeting efficiency is affected by tumor heterogeneity and individual differences. Optimizing pharmacokinetics and pharmacodynamics is also a critical issue. Furthermore, the production process is complex and requires stringent quality control, and regulatory approval is quite rigorous. Overcoming these obstacles requires in-depth basic research, systematic clinical trials, and advancements in technology and processes to fully realize the potential of nanomedicines in treating head and neck cancers.

## 5 Conclusion

While aggressive treatment modalities such as inhibitors, chemotherapy, radiotherapy, and surgical resection have been extensively employed in the clinical management of HNC, these approaches have yielded limited efficacy, with patients often experiencing negligible improvements. Over the past few decades, there has been a concerted effort to gain a comprehensive understanding of the etiology of HNC, resistance mechanisms, and the evolving nature of the disease. This research encompasses the examination of well-established targets such as the PI3K/AKT/mTOR pathway and EGFR signaling, among others. Additionally, there is a growing recognition of the necessity for further exploration of novel targets, including MET signaling and Notch signaling.

Presently, nanomaterials are being prominently utilized in the realm of nanomedicine due to their exceptional properties, particularly in the context of cancer detection and therapy. In the context of HNC treatment, nanomaterials have demonstrated the capacity to augment the effectiveness of chemotherapy while minimizing associated toxicities. The utilization of nanoparticles as carriers for drug delivery in HNC has opened up avenues to alleviate patient suffering and extend the lives of those with advanced-stage disease. This therapeutic approach has garnered substantial attention within the scientific community.

In recent times, a multitude of nanoscale materials with specialized characteristics have been identified for deployment as targeted drug delivery systems. Although the majority of nanoparticle-based targeted delivery methods have not yet transitioned into routine clinical practice, they hold significant promise for the treatment of HNC. It is our contention that the application of nanomaterial-based chemotherapy will not only enhance the diagnosis, treatment, and prevention of HNC but will also have a profound impact on human health in the foreseeable future, as research on nanomaterials and the advancement of HNC treatments continue to evolve. In the future, we should actively explore new types of nanomaterials to find those with better biocompatibility, higher targeting efficiency, and improved stability, thereby enhancing therapeutic efficacy and reducing side effects. Additionally, optimizing drug delivery systems is crucial, which includes improving the design of nanoparticles to enhance their ability to control drug release within the body and ensure precise delivery to the target site. Lastly, conducting large-scale clinical trials is essential to validate the safety and efficacy of nanomedicines through extensive clinical data, thereby establishing a solid foundation for their clinical application. These measures will help advance the use of nanomedicines in medicine, particularly in breakthroughs in cancer treatment.
